# From the circular economy to the sustainable development goals in the European Union: an empirical comparison

**DOI:** 10.1007/s10784-021-09553-4

**Published:** 2021-11-01

**Authors:** José Miguel Rodríguez-Antón, Luis Rubio-Andrada, María Soledad Celemín-Pedroche, Soraya María Ruíz-Peñalver

**Affiliations:** 1 Business Administration Department, University Autonomous of Madrid, Carretera de Colmenar Viejo, K. 15, 28049 Madrid, Spain; 2 Department of Applied Economics, University Autonomous of Madrid, Carretera de Colmenar Viejo, K. 15, 28049 Madrid, Spain; 3 Department of Applied Economics, University of Granada, Avda. del Hospicio, s/n, 18071 Granada, Spain

**Keywords:** Circular economy, Sustainable development goals, Sustainability, 2030 agenda, European Union

## Abstract

The European Union (EU) is trying to accelerate the transition from the current linear economy to a circular economy (CE). In fact, the CE is considered a tool to attain sustainable development goals (SDGs). In this sense, this paper aims at analysing the interaction between the CE and SDGs in the context of the new 2030 Agenda and the European CE strategy; thus contributing to the scarce empirical literature that links the potential of the European CE strategy to the achievement of the SDGs set by the 2030 Agenda. Three specific research questions have been formulated. First, could the objectives defined in the 2030 Agenda be considered homogeneous, and could they uniquely measure the concept of sustainability? Second, are there significant correlations between the implementation of a CE in the EU and the SDGs? Finally, is the behaviour of the 28 countries that make up the EU homogeneous in terms of the results of the initiatives aimed at the implementation of a CE? From these questions, nine hypotheses are put forward concerning the possible relationships between a CE implementation and the fulfilment of SDGs in the EU. Using a correlation analysis, an exploratory factor analysis, and a cluster analysis, it has been demonstrated that (a) SDGs do not univocally measure the concept of sustainability; (b) there are significant relationships between CE and SDGs in the EU; (c) the behaviour of these European countries is not homogeneous.

## Introduction

In the twentieth century, the world began to think that the economic development could put environmental sustainability at risk. This global feeling led to the Earth Summits—organized since 1972 with the help of the United Nations as meetings of world leaders—to help define ways to stimulate sustainable development at a global level. This concern was the germ of the Brundtland Report, carried out for the United Nations by the Brundtland Commission in 1987. The term ‘sustainable development’ was used for the first time in this report, which aimed to rethink economic development policies because of their high environmental cost. As a continuation of these and other environmental activities, projects, and policy recommendations, the world’s most important supranational institutions have been concerned with ensuring the sustainability of the planet since the beginning of this century. In 2000, the United Nations approved the ‘Declaration of the Millennium’ (https://www.preventionweb.net/files/13539_13539ARES552ResolutiononUNMillenniu.pdf), intended to protect humans and ensure international relations. Fifteen years later, this organisation approved the ‘2030 Agenda for Sustainable Development’ (https://undocs.org/en/A/RES/70/1), which is a set of targeted measures not only for humans but also for the planet in general (Rodriguez-Anton et al., 2019).

However, this effort to achieve sustainability for the planet has been carried out not only by this international organisation, but the EU has also tried to boost the sustainability of the European space in an attempt to accelerate the transition from the linear economy to a CE as much as possible (Rodríguez-Antón, 2019; Rodríguez-Antón & Alonso-Almeida, 2018).

After the European Commission issued the communication entitled ‘Roadmap to a Resource Efficient Europe’ (https://eur-lex.europa.eu/legal-content/EN/TXT/PDF/?uri=CELEX:52011DC0571&qid=1618665628781&from=EN) in 2011, Europe has continued trying to increase sustainability. In 2015, the European Union agreed to ‘Close the Circle: An Action Plan of the European Union for the Circular Economy’ and asked for a commitment to implement a CE in the member states, regions, cities, companies, and citizens. A year later, in 2016, the European Commission approved the European Action for Sustainability (https://eur-lex.europa.eu/legal-content/EN/TXT/PDF/?uri=CELEX:52016DC0739&from=ES), which also reaffirms the EU's commitment to these sustainability goals.

These communications were followed by others such as ‘A European Strategy for Plastics in a Circular Economy’ [SWD (2018) 16 final] (https://eur-lex.europa.eu/resource.html?uri=cellar:2df5d1d2-fac7-11e7-b8f5-01aa75ed71a1.0001.02/DOC_1&format=PDF), a plastic-oriented communication; ‘A Sustainable Bioeconomy for Europe: Strengthening the Connection Between Economy, Society and the Environment’ [COM (2018) 673 final] (https://eur-lex.europa.eu/legal-content/EN/TXT/PDF/?uri=CELEX:52018DC0673&qid=1618666423553&from=EN); ‘Environmental Implementation Review 2019: A Europe that protects its citizens and enhances their quality of life’ [COM (2019) 149 final] (https://ec.europa.eu/environment/eir/pdf/eir_2019.pdf); and a reflection paper published in 2019 entitled ‘Towards a Sustainable Europe by 2030’ (https://op.europa.eu/en/publication-detail/-/publication/3b096b37-300a-11e9-8d04-01aa75ed71a1/language-en/format-PDF).

At the end of that year, the Commission sent to the European Parliament, the council, the European economic and social committee, and the committee of the regions the communication entitled ‘The European Green Deal’, which ‘aims to transform the EU into a fair and prosperous society, with a modern, resource-efficient and competitive economy where there are no net emissions of greenhouse gases in 2050 and where economic growth is decoupled from resource use’ (https://eur-lex.europa.eu/resource.html?uri=cellar:b828d165-1c22-11ea-8c1f-01aa75ed71a1.0002.02/DOC_1&format=PDF). This communication was accompanied by a major European investment plan to promote the Green Deal at the beginning of 2020 (Rodríguez-Antón & Alonso-Almeida, 2021).

Finally, we find the communication sent on March 11, 2020, by the Commission to the European Parliament, the council, the European economic and social committee, and the committee of the regions entitled ‘A new Circular Economy Action Plan. For a cleaner and more competitive Europe’. This report “provides a future-oriented agenda for achieving a cleaner and more competitive Europe in co-creation with economic actors, consumers, citizens and civil society organisations” (https://eur-lex.europa.eu/resource.html?uri=cellar:9903b325-6388-11ea-b735-01aa75ed71a1.0017.02/DOC_1&format=PDF).

In addition to these reports, the EU has approved a series of directives and regulations between 2018 and 2019 with a clear impact on the implementation of a CE in the European space. These are listed in Table 1.

**Table 1:** EU Directives and Regulations on the CE (2018–2019)

Directives	
Directive (EU) 2018/849 amending Directives 2000/53/EC on end-of-life vehicles, 2006/66/EC on batteries and accumulators and waste batteries and accumulators, and 2012/19/EU on waste electrical and electronic equipment	
Directive (EU) 2018/850 amending Directive 1999/31/EC on the landfill of waste, the Directive (EU) 2018/851 amending Directive 2008/98/EC on waste	
Directive (EU) 2018/852 amending Directive 94/62/EC on packaging and packaging waste	
Directive (EU) 2019/883 on port reception facilities for the delivery of waste from ships, amending Directive 2010/65/EU and repealing Directive 2000/59/EC	
Directive (EU) 2019/904 on the reduction of the impact of certain plastic products on the environment, and the Directive (EU) 2019/771 on certain aspects concerning contracts for the sale of goods, amending Regulation (EU) 2017/2394 and Directive 2009/22/EC, and repealing Directive 1999/44/EC	
Regulations	
Regulation (EU) 2019/1009 laying down rules on the making available on the market of EU fertilising products and amending Regulations (EC) No 1069/2009 and (EC) No 1107/2009 and repealing Regulation (EC) No 2003/2003	
Regulation (EU) 2019/424 laying down ecodesign requirements for servers and data storage products pursuant to Directive 2009/125/EC of the European Parliament and of the Council and amending Commission Regulation (EU) No 617/2013	
Regulation (EU) 2019/1784 laying down ecodesign requirements for welding equipment pursuant to Directive 2009/125/EC of the European Parliament and of the Council	
Regulation (EU) 2019/2021 laying down ecodesign requirements for electronic displays pursuant to Directive 2009/125/EC, amending Regulation (EC) No 1275/2008 and repealing Regulation (EC) 642/2009	
Regulation (EU) 2019/2023 laying down ecodesign requirements for household washing machines and household washer-dryers pursuant to Directive 2009/125/EC, amending Regulation (EC) No 1275/2008 and repealing Regulation (EU) No 1015/2010	
Regulation (EU) 2019/2019 laying down ecodesign requirements for refrigerating appliances pursuant to Directive 2009/125/EC and repealing Regulation (EC) No 643/2009	
Regulation (EU) 2019/2024 laying down ecodesign requirements for refrigerating appliances with a direct sales function pursuant to Directive 2009/125/EC	
Regulation (EU) 2019/2022 laying down ecodesign requirements for household dishwashers pursuant to Directive 2009/125/EC amending Regulation (EC) No 1275/2008 and repealing Regulation (EU) No 1016/2010	

Table one shows the main directives and regulations enforced by the EU to promote CE. Source: Calisto Friant et al. (2021)

The EU wants the CE to be a tool to achieve most of the 17 sustainable development goals (SDGs) (Rodríguez-Antón et al., 2019), as confirmed by the ‘Communication from the commission to the European parliament, the council, the European economic and social committee, and the committee of the regions. Next steps for a sustainable European future. European action for sustainability’ {SWD, 2016 390 final} (https://eur-lex.europa.eu/legal-content/EN/TXT/PDF/?uri=CELEX:52016DC0739&from=ES), where it is stated that ‘The circular economy (SDG 6, 8, 9, 11, 12, 13, 14, 15) offers a transformative agenda with significant new jobs and growth potential and stimulating sustainable consumption and production patterns’, (pg. 8) or that ‘The transition to the circular economy offers a chance for Europe to modernise its economy, making it more future proof, green and competitive’ (pg. 8). Besides, in the aforementioned European Action for Sustainability, the European Commission reaffirms its commitment to sustainable development. This report explains the actions that the EU is taking to fulfil the SDGs.

Consequently, the analysis of the guidelines, recommendations, and reflections emanating from the EU seems to indicate the existence of a relationship between a CE and compliance with the SDGs, a claim that is supported by previous studies carried out by the academy and will be demonstrated in the literature review. Therefore, three research questions have been formulated related to the SDGs and circular economy in the European Union. To obtain answers to these research questions, a correlation analysis, an exploratory factor analysis, and a cluster analysis will be carried out.

This paper is structured as follows: after the introduction, the literature review will be presented, followed by the research questions and hypotheses. The research methods and sample will then be analysed, and thereafter, the results will be provided and analysed to lead to the final conclusion.

## Literature review

In 2015, the SDGs replaced the Millennium Development Goals (MDGs) and have been demonstrated to be a much-improved version of the MDGs. The 2030 Agenda promotes greater convergence towards sustainability, and it also strengthens other social aspects, such as human rights, equity or social justice (Kumar et al., 2016), and environmental sustainability, which were relegated despite the fact that Mebratu (1998) showed that sustainability cannot be reduced to the combination of these three dimensions. Instead, economic sustainability depends on social sustainability, and these two depend on the environmental one. All these improvements were implemented in the Agenda 2030 through 17 SDGs and their correspondent targets and indicators, which present a more integrated structure than the MDGs, according to the three dimensions of sustainability (economic, social, and environmental spheres; UN ESC, 2017). These goals, targets, and indicators define a network with a great number of interconnections among the different thematic areas (Dantas et al., 2021).

Nevertheless, the Agenda 2030 is reprimanded for several aspects. SDGs were criticised for not having a comprehensive sustainable development theory, being supported by weak theoretical foundations, or not establishing priorities among the targets (Spasier et al., 2017). Curiously, one of the great criticisms is associated with the interrelations between goals and targets. Spasier et al. (2017) unveiled an important inconsistency between the SDGs, underlining that economic growth is compatible with socioeconomic goals whilst simultaneously prejudicing environmental ones. These authors assert that the ‘End of Poverty’ involves good indicators—the ‘Social Inclusion’ pillar is much weaker with high and low factor loadings, and the ‘Environment’ pillar is the worst defined with its indicators showing a weak link. Pradhan et al. (2017) also observed that SDGs related to increase human development and socioeconomic improvements were conflicting with environmental goals. For instance, SDGs 1, 3, 4, 10, 12, and 13 have synergetic relationships, but the same analysis indicates negative correlations between SDG 1 and SDGs 7, 8, 9, and 15 (Pradhan et al., 2017, p. 1171). These results could raise the question, whether the goals of the 2030 Agenda are suitable to measure the concept of sustainability. Anyway, the current SDGs and sustainable development are long-term paradigms, hence they are subject to unexpected changes that can modify them or even alter future outcomes. For this reason, current analyses—showing conflicts among some SDGs and not considering future trends–must find solutions to the present SDGs and the sustainability process within the existing capacities available (Spasier et al., 2017). Finally, there are authors that even consider adding other pillars to the current sustainable development paradigm. Huttmanová et al. (2019a) suggest adding the institutional dimension, while Prieto-Sandoval et al. (2017) state that newer visions show sustainable development having a fifth pillar, ‘time’, since actions carried out towards sustainability exert an impact in the short, medium, and long term.

According to the European CE, strategy is considered an innovative school of thought in sustainable development, but it is still in its infancy (Murray et al., 2017). However, its roots go back to the earlier work of Pearce and Turner (Sacchi et al., 2018), and even countries such as China implemented this paradigm in their economies several decades ago. Overall, the European CE concept tries to decouple economic growth from resources depletion, encouraging waste decrease in a transition from the ‘cradle-to-grave’ (linear economy) mindset towards the ‘cradle-to-cradle’ process (circular one; Gregson et al., 2015). In this sense, the factors that guarantee the development of circular economy in a model on economic growth at the European level are ‘renewable energy, productivity of the resources, recycling rate, environmental employment and innovation’ (Busu, 2019, p. 10). The European CE strategy implies great challenges for socioeconomic stakeholders, especially for businesses, which must assume important risks to transition from the linear economy to an innovative circular one. However, if firms surpass these risks, business will be more competitive in markets (Jørgensen & Remmen, 2018). The implications of the CE strategy on firms justify the great variety of publications focused on the business concept of a CE and its implementation in firms (Merli et al., 2018).

Nevertheless, there is a lack of consensus on the definition of CE. Korhonen et al. (2018) stated that the European CE definition is superficial and unorganised, an amalgam of ideas from different scientific fields including industrial ecosystems, industrial ecology, material flows, economy, biology, environmental economics, etc. Other authors (Lewandowski, 2016; Lieder & Rashid, 2016; Sacchi et al., 2018) reviewed the diverse existing concepts of CE in their various acceptations. All these authors asserted that certain aspects of CE—even institutional, cultural, or legislative issues—are missing in the literature. Murray et al. (2017) have also criticised the current CE approach for: firstly, not including the social dimension, crucial for sustainability, and secondly, planning weakly-based superficial goals and not foreseeing the future consequences of its implementation.

Despite the limitations of CE, the current concept has two main contributions. First, CE recovers the importance of the material life cycle, its value, and its quality. Second, CE offers the possibilities of a sharing economy alongside sustainable production for more suitable production-consumption patterns (Korhonen et al., 2018), through CE business models such as slowing the loops (e.g. satisfying needs without the ownership of a product, extending product value, designing long-life products, encouraging sufficiency, or prolonging product life at the end-user level) or closing the loops (e.g. extending resource value or industrial symbiosis; Bocken et al., 2016).

In order to contrast the relationship between a CE and SDGs, a literature review was carried out to explore the state of the art of academic research on the topic, both globally and in the EU. To achieve this aim, a search was undertaken in January 2021 using Web of Science (WoS) and Scopus, the most important scientific databases (Aghaei-Chadegani et al., 2013), as well as Google Scholar, to complement the literature review.

To avoid biases in the literature search, the following keywords were used: ‘circular economy AND sustainable development goals’, ‘circular economy AND 2030 Agenda’, and ‘circular economy AND sustainability goals’. To cover the largest number of related publications and not underestimate the grey literature (e.g. contributions to the CE from the Ellen MacArthur Foundation), neither chronological limits nor other restrictions were applied (such as language, type of document, etc.). In WoS, the ‘Core Collection’ and the advance search were selected, and the keywords mentioned above were employed in the search criteria ‘Title, ‘Abstract’ and ‘Keywords’. In Scopus, the same keywords and search criteria were used. WoS returned 299 results and Scopus supplied 517 documents. As mentioned, these searches were complemented by material found in Google Scholar. In the latter case, the publications obtained were previously collected in WoS or Scopus, and hence the duplicate material was dismissed. As a result, a total of 816 publications was found, of which 235 were overlapping articles between WoS and Scopus and were deleted. Thus, the total number of documents was 581.

After the search process, the collected material was organised in a database, which implied homogenising the bibliographic cataloguing fields employed by WoS and Scopus. Overall, the organisation and review of the material showed the transversality and dispersion of the topic in fields such as environmental science, economics, industrial ecology, or education, among other disciplines. A large portion of the 581 documents was ignored for the literature review as all the references were related to CE and sustainability but the vast majority made contributions to stimulate the sectoral transition to more circular and sustainable production models (e.g. improvements in production processes, energy consumption, bioenergy, innovative design, supply chains, efficiency of resources, use of alternative materials, etc.). Other documents analysed how to implement a CE, consumer perception, and waste management and treatment, among other very revealing approaches, but were quite far from the goal of this paper, namely analysing the relationship between a CE and SDGs. Almost 60 works related to the topic were found. Despite the recently growing number of scholars who consider a CE an instrument to achieve SDGs (Holden et al., 2017; Rodríguez-Antón, 2019; Rodríguez-Antón et al., 2019; Sauvé et al., 2016; Schroeder et al., 2018; Xue et al., 2010; etc.), the literature review confirmed that the studies linking the potential of the European CE strategy to the achievement of SDGs set by the 2030 Agenda are still relatively scarce.

Nevertheless, there are authors who state that the role of CE in achieving the SDGs is yet quite questioned among scholars (Suárez-Eiroa et al., 2019). Sauvé et al. (2016) distinguished two research trends that would justify why there is no clarity on the topic. On the one hand, there are authors who view sustainable development as a set of failed initiatives that were implemented for a linear system, for which CE offers a solution. On the other hand, there are authors who assume that CE and sustainable development are consistent and even interdependent disciplines. These authors also indicate that CE can be considered a tool to achieve sustainability. These discrepancies are due, among other things, to the CE paradigm that arose in a critical environmental context with great impact on all areas of society (European Commission, 2015) and thus producing an array of related opinions. Other reasons that explain the disparity in opinions are explained by the ‘novelty’ of the 2030 Agenda and the European CE strategy, both of which were signed in 2015. Additionally, there is still no consensus on the acceptance of the current theoretical framework of CE (Kirchherr et al., 2018; Korhonen et al., 2018; Prieto-Sandoval et al., 2018). Finally, many stakeholders, especially businesses, find it difficult to effectively implement the principles of circularity (McDowall et al., 2017; Pauliuk, 2018). All these inconveniences imply that research concerning CE and SDGs is still in its infancy and will increase in the following years as both paradigms evolve.

Regarding the scope of these research studies, most of them are theoretical contributions. Many of them analyse CE and its implementation to achieve sustainability (Kassenberg, 2017; Prieto-Sandoval et al., 2017; Raftowicz, 2016; Suárez-Eiroa, et al., 2019), highlighting the challenges it implies. Other studies perform inductive analysis based on practical cases of CE implementation to justify its importance in achieving the SDGs (Pla-Julián & Guevara, 2019; Ribeiro et al., 2017). We must also underline the increase of research providing indicators to evaluate the integration of CE principles and sustainability (Fioramonti et al., 2019; Kravchenko et al., 2020; Muñoz-Torres et al., 2018). Despite most publications referring to the principles and postulates of CE and sustainability, some of them are focused on economic activities–such as Boluk et al. (2019) analysing tourism, or Buch et al. (2018) and Pigosso and McAloone (2017) dealing with the role of science and universities in the transition process towards a circular economy and sustainable development.

Some researchers stress the strong correlation between the principles of CE and SDGs (Pla-Julián & Guevara, 2019; Rodríguez-Antón, 2019; Rodríguez-Antón et al., 2019; Schoreder et al., 2018). Pla-Julián and Guevara (2019) indicate the synergies associated with CE as an alternative model of value creation in the productive system, with positive externalities in the social and environmental spheres. After studying several examples of businesses that are developing or are going to implement CE initiatives, these authors conclude that CE should be a crucial element to reach the economic scope of sustainability, indicating the direction of the necessary structural changes to guarantee businesses’ transition to a circular productive system.

Following this idea, Suárez-Eiroa et al. (2019) propose a series of operational principles to link the theoretical objectives of the CE to SDGs, and hence facilitate the practical implementation of both strategies. These principles are synthesised in Suárez-Eiroa et al., (2019, p. 957):

1. *Adjusting inputs to the system to regeneration rates* This principle applies minimisation and even elimination strategies for non-renewable inputs and their replacement with renewable resources.
2. *Adjusting outputs from the system to absorption rates* As in the previous principle, this second one consists of reducing and eliminating the waste generated in the production system that cannot be recovered or reintegrated.
3. *Closing the system* This links the stages of waste management and acquisition of resources to reduce the consumption of raw material.
4. *Maintaining resource value within the system* This operation principle is based on two axes: (a) improving the durability of products, avoiding obsolescence (scheduled or not) since it is one of the main problems of product replacement; and (b) encouraging the introduction of resources throughout the different stages of production (through reuse, repair, restoration, conditioning, etc.).
5. *Reducing the system’s size* This principle was also identified by Paulik (2018) to reduce the volume of resources used. For this, it is necessary to reduce the demand for products, as well as to replace the production and consumption of non-sustainable products with sustainable ones.

Principles (3) to (5) are considered the core of this proposal since they are crucial to achieving the theoretical objectives of the CE and SDGs. The principles (6) Design for the CE and (7) Educate for the CE, are considered to be transversal to the previous principles. They are based on the sustainable design of products (ecodesign, packaging, product optimisation, durability, etc.) and on citizen education to protect the natural environment and conserve available resources (Suárez-Eiroa et al., 2019, p. 956).

Schoreder et al. (2018) explore the synergies obtained from CE practices for the implementation of the SDGs. Although this research was carried out in developing countries, their analysis can be extended to the EU with the appropriate adaptations. These authors found strong relationships between the circularity practices carried out and the SDGs set out in the 2030 Agenda. To illustrate this, the following five categories are used to describe the existing relationship: (1) direct or strong relationship of CE practices with achieving an SDG; (2) indirect relationship (the link is established through other SDGs); (3) progress in the SDGs reinforces the adoption of CE practices—this category indicates that an SDG from the list of SDGs has an inverse causality with the CE; that is, instnityead of CE initiatives contributing to attaining an SDG, achieving an SDG will contribute to the implementation of more CE practices–. Continuing the description of categories, these authors look for; (4) a weak or non-existent relationship; and (5) an opportunity for cooperation to promote CE practices. Overall, their results show a strong direct relationship between various CE practices and SDGs 6, 7, 8, and 12 (category 1), while SDGs 1, 2, and 14 are indirectly related to CE initiatives (category 2). On the other hand, SDGs 4, 9, 10, 13, 16, and 17 should be included in category 3. Finally, SDGs 3, 5, 10, 11, and 16 show a weak or null relationship (category 4). Schroeder et al. (2018) included target 16 in two categories of analysis. This is due to the analysis being carried out by ‘targets’, and in this case, the relations between them and the CE initiatives have been equally divided between categories (3) and (4).

Schroeder et al. (2018), Silvestre and Ţîrcă, (2019), and Hidayatno et al. (2019) also support that sustainable development cannot be reached without innovation and technological progress, emphasising the importance of the ‘Industry 4.0’ (I4.0 business model related to firms that incorporate new and innovative technologies) as the most suitable business model to achieve SDGs. In addition, Schroeder et al. (2018) indicate that I4.0 must focus on enhancing the CE and achieving SDGs. In this sense, Dantas et al. (2021) demonstrate how the CE and I4.0 practices contribute to the attainment of SDGs. Their findings show that a combination of CE and I4.0 practices can contribute to achieving SDGs 7, 8, 9, 11, 12, and 13.

On the other hand, there are studies that highlight the numerous challenges related to the CE paradigms and sustainable development, including research that underscores the incompatibility between these paradigms and economic growth or publications that emphasise implementation problems that inhibit the use of CE as a tool to achieve SDGs in its current application. For instance, Kirchherr et al. (2017) compared 114 definitions of CE and concluded that only 11% included concepts of sustainable development and only 13% covered the three dimensions of sustainability (environmental, social, and economic spheres). Korhonen et al. (2018) reviewed different concepts of CE related to sustainable development and found six limitations associated with the CE concept and environmental sustainability, justifying that there is a need to analyse the concept of CE from the perspective of sustainable development. Millar et al. (2019) indicate that the theoretical association between CE and sustainable development has not been properly established and that there are failures in addressing many of the issues of the CE, for which the linear economy was severely criticised. Their arguments are supported not only by the multitude of divergent definitions that limit the capability of CE to achieve sustainable development, but also by the biophysical barriers of CE that question whether it can maintain economic growth without causing the environment deterioration. Following Korhonen et al. (2018), Millar et al. (2019) believe that the current conception of CE does not clarify how CE can promote social equity or how social profits can be measured. Overall, most criticisms against CE as a driver to achieve SDGs underline that although CE could be more sustainable than the current linear economy from an environmental perspective, it could have the same environmental impact in the long term.

From the literature review, it follows that more research is needed in the following subjects: (a) The homogeneity of the SDGs regarding their ability to measure the concept of sustainability and its evolution due to the potential changes (e.g. new technologies, social advances, etc.) influencing the goals, targets, and indicators; (b) The current existence of significant correlations between a CE implementation in the EU and SDGs; and (c) The EU behaviour homogeneity in the CE-implementation initiatives’ results.

## Research questions and hypotheses

From the comparative analysis of the relationships between the CE and SDGs included in the introduction and the discussion of recent relevant literature above several research questions are raised and a set of hypotheses must be contrasted.

With regard to the former, the objective is to answer the following three research questions:

RQ1: Can the 2030 Agenda objectives be considered homogeneous, and can they adequately measure the concept of sustainability?
RQ2: Are there significant correlations among the indicators of a CE implementation in the EU and the 17 SDGs?
RQ3: Is the behaviour of the 28 EU countries homogeneous in terms of the CE implementation initiatives’ results in this space?

In the same way, the hypotheses to be tested derived from the analysis of the previous literature and the European regulations and recommendations regarding CE explores the existing relationships among the CE and each SDG. Although since 2016 the communications have continuously stated how the effective implementation of a CE model could facilitate compliance with the SDGs, the clearest relationship between the two concepts is reflected on page 10 of the European Action for Sustainability, which indicates that CE and SDGs 6, 8, 9, 11, 12, 13, 14, and 15 are related. On the other hand, the previous literature includes the following: a) in Schoreder et al. (2018), there is a strong direct relationship between CE practices and SDGs 6, 7, 8, 12, and 15, and an indirect relationship with SDGs 1, 2, and 14, thus showing that there is a relationship, with uncertain meaning, with other SDGs; and b) in Rodríguez-Antón et al. (2019), there is a significant relationship between CE and SDGs 2, 3, 5, 8, 9, 10, 11, 12, 13, 14, and 16.

Consequently, from the relationships detected in these studies under consideration (Rodríguez-Antón et al., 2019; Schoreder el al., 2018) and in the European Commission communications, the following hypotheses are presented, which appear, at least, in two of these groups of references:

H1. The CE and SDG 2 are related.

H2. The CE and SDG 6 are related.

H3. The CE and SDG 8 are related.

H4. The CE and SDG 9 are related.

H5. The CE and SDG 11 are related.

H6. The CE and SDG 12 are related.

H7. The CE and SDG 13 are related.

H8. The CE and SDG 14 are related.

H9. The CE and SDG 15 are related.

## Research methods and sample

Based upon the study conducted by Rodríguez-Antón et al. (2019), an attempt has been made to deepen the study of the relations between the CE in the EU and the level of achievement of the SDGs in that European space. For this, the following research methods have been used:

- Both the variables considered in the SDG Index and Dashboards Report 2018 (Sachs et al., 2018) have been deeply analysed to measure compliance with the SDGs. Some of them act in a positive sense (the higher the value, the greater the degree of compliance with an SDG) and others in the negative sense (the higher the value, the lower the level of compliance with an SDG). Likewise, some CE indicators used by the EU act in a positive sense (the higher the value, the greater the degree of alignment with the CE) and others in the negative sense (the greater the value, the lower the degree of alignment with the CE).
- The correlations between the 17 SDGs have been analysed.
- The relationships between the CE and SDGs have been thoroughly studied, based on an analysis of the existing literature and the international legislation approved by the United Nations and the EU concerning SDGs and the CE. This has allowed a series of hypotheses to be formulated and subsequently tested.
- For the 28 EU countries, the level of compliance with the 17 SDGs was analysed for the year 2017. Based on the transformed data from the SDG Index and Dashboards Report 2017 (Sach et al., 2017) exploratory factor analysis (EFA) was performed to calculate the relationship between CE and SDGs, as well as how many components these goals have.
- A cluster analysis was run to identify how the EU nations are grouped, using the main CE indicators to see their relationship with the fulfilment of the SDGs.
- Among the 28 EU members, EFA has been conducted with a selection of CE indicators calculated by Eurostat to find out how many components these indicators have.
- Next, the correlations between all these CE indicators and the SDGs were discovered.
- Finally, from these results, a discussion was held, the three research questions were answered, and the nine hypotheses were contrasted, thereby reaching engaging conclusions.

## Results

For the analysis of the variables that make up each SDG, we have started from a study of the methodology used by Sachs et al. (2018) to calculate the degree of compliance by each country with the SDGs. Specifically, 88 global indicators and 111 indicators for Organization for Economic Co-operation and Development (OECD) countries were used in that report. However, within most of the SDGs, some indicators are defined in such a way that the higher the value, the lower the degree of compliance with that SDG. For example, in SDG 12, the indicator or variable 3, called production-based SO2 emissions, should be considered as being in the opposite direction to its value; that is, the higher the value of that variable, the further that country will be from compliance with that SDG. So that all the indicators work in a positive sense (the greater, the better), the inverse of the indicator has been carried out when necessary, those defined in such a way that the greater the value, the lower the compliance with that SDG. Appendix 1 shows the variables/indicators that could be used because there is enough information from the 28 EU countries (all variables for which eight or more values were missing—more than 30% of the population—or values that were equal in all cases have been eliminated), as well as the direct or inverse sense of the variable.

For the analysis of CE initiatives, the source of quantitative information used has been the Eurostat monitoring framework, which considers four groups of indicators with a total of nine indicators. In this case, only one indicator—generation of municipal waste per capita—acted in the opposite direction; that is, the higher the generation of municipal waste, the further a country is from reaching a CE (see Appendix 1).

As shown in Appendix 2, the 17 SDGs considered by the UN in the 2030 Agenda are not independent of one another. On the contrary, there are significant correlations between some of them. For example, SDG 1 is correlated with SDGs 3, 8, 13, and 16; SDG 2 with SDG 6; SDG 3 with SDGs 5, 6, 8, 9, 11, 12, 13, 14, 15, 16, and 17, and so on. However, a large part of the SDGs is not correlated with others. For example, SDG 2 is only related to SDG 6, so it seems that they aim for different objectives. In order to check whether the 17 SDGs have a unique direction and there is a single general objective of sustainability, an exploratory factor analysis (EFA) has been carried out with the 17 SDGs, which has given a Kaiser–Meyer–Olkin sample adequacy measure of 0.637, a Bartlett’s test of sphericity with a chi-square of 351.5 with 136 degrees of freedom and a degree of significance of 5.33553E-21. From this factor analysis, and after performing a Varimax rotation with Kaiser, it has been found that there are five factors that explain 78.965 percent of the total variance.

The first of these five factors, that we call SDGI1 (Sustainable Development Goal Index), explains 26.479 of the total variance and consists of seven of the 17 SDGs, namely SDGs 3, 6, 10, 11, 12, 15, and 17. The second factor (SDGI2) is composed by SDGs 4, 5, 7, 8, 9, and 16, and the third factor (SDGI3) is for SDG 13 and 14—the latter with a negative sign, which seems to indicate that high protection of the marine environment may be counterproductive to the sustainability of the planet while multiple resources are obtained from the sea. The fourth factor (SDGI4) represents only SDG 1, and the fifth factor (SDGI5) represents only SDG 2 with a negative sign, which may reflect that in order to reach the goal of zero hunger, it will be necessary to engage in a huge consumption of resources, which could jeopardise the sustainability of the planet (see Table 2).

**Table 2:** Matrix of Rotated Components of SDGs

	Component				
	1	2	3	4	5
SDG1				**0.885**	
SDG2					** − 0.903**
ISDG3	**0.708**	0.389	0.375		
ISDG4		**0,756**		0.442	
ISDG5		**0.908**			
ISDG6	**0.677**				0.514
ISDG7		**0.664**	0.418	− 0.443	
ISDG8	0.578	**0.630**			
ISDG9	0.612	**0.717**			
ISDG10	**0.487**		0.342		0.336
ISDG11	**0.766**	0.329			
ISDG12	** − 0.736**				
ISDG13			**0.827**		
ISDG14			** − 0.813**		
ISDG15	**0.751**				− 0.356
ISDG16	0.608	**0.618**		0.370	
ISDG17	**0.636**	0.321	− 0.418		

Extraction method: Principal component analysis. Rotation method: Varimax normalization with Kaiser (the rotation has converged in 10 iterations). *Bold means that correlation is significant at the 0.01 or 0.05 bilateral level

Once the EFA corresponding to the 17 SDGs had been carried out, the same analysis was performed now applied to the 9 variables that define the degree of EU compliance with the CE. It has been verified that there are three factors that explain 84.775 percent of the total variance. The first one, which we name CEI1 (Circular Economy Indicator), explains 34.345 percent of it, while the other two explain, respectively, 26.614 percent (CEI2) and 23.816 percent (CEI3).

The first of these factors, CEI1, comprises the generation of municipal waste per capita (CEV1), the recycling rate of overall packaging (CEV3), the recycling of biowaste (CEV5), the circular material use rate (CEV7), and persons employed (CEV9), these last two with a negative sign. The second factor, CEI2, is integrated by the recovery rate of construction or demolition waste (CEV6) and gross investment in tangible goods (CEV8). Finally, the third factor, CEI3, is formed by the recycling rate of municipal waste (CEV2) and the recycling rate of e-waste (CEV4) (see Table 3).

**Table 3:** Matrix of Rotated Components of CE Variables

	Component		
	1	2	3
CEV1	**0.566**	− 0.354	0.319
CEV2			**0.972**
CEV3	**0.914**		
CEV4			**0.972**
CEV5	**0.914**		
CEV6		**0.962**	
CEV7	** − 0.733**	0.431	
CEV8		**0.962**	
CEV9	** − 0.733**	0.431	

Extraction method: Principal component analysis. Rotation method: Varimax normalization with Kaiser (the rotation has converged in 5 iterations. *Bold means that correlation is significant at the 0.01 or 0.05 bilateral level

Since there is no single factor that satisfactorily explains the concept of CE, but there are the three factors just indicated, a study of the correlations among each of the 17 SDGs and the three factors of CE considered was conducted. As shown in Appendix 3, there are abundant highly significant correlations (for a level of 0.01 bilateral significance) between both groups of variables. Specifically, there are significant correlations among SDGs 3, 6, 7, 8, 9, 10, 11, 12, 13, 14, 15, 16, and 17 with all three CE factors. If we focus only on the first factor, it is very highly correlated with SDGs 6, 8, 9, 11, 16, and 17 at a level of significance of bilateral 0.01 and it is correlated with SDGs 3 and 10 at a level of significance of 0.05 bilateral. The second factor is strongly correlated with SDGs 3, 6, 10, and 15—all of them with a negative sign—and with SDG 12 at a level of significance of bilateral 0.05. Finally, the third factor is correlated at a level of significance of bilateral 0.05 with SDGs 7 and 14, and in the latter case, with a negative sign.

Once the correlations between the CE and the SDGs have been analysed, a cluster analysis using the nine CE indicators was performed in order to find out whether the results obtained by the 28 EU nations are homogeneous in terms of the implementation of a CE in the region. The cluster results, obtained at two stages—the first using Ward’s hierarchical method and the second using K-means –, showed that there are three groups of countries in the EU (see Table 4).

**Table 4:** Cluster analysis results

Cluster		CEV1INV	CEV2	CEV3	CEV4	CEV5	CEV6	CEV7	CEV8	CEV9
1	Average	**1.091**	**0.238**	**1.205**	**0.238**	**1.205**	− 0.362	− 0.879	− 0.362	− 0.879
2	Average	− 0.393	**0.532**	− 0.433	**0.532**	− 0.433	**0.598**	**0.520**	**0.598**	**0.520**
3	Average	− 0.516	− 1.260	− 0.573	− 1.260	− 0.573	− 0.696	**0.039**	− 0.696	**0.039**
Total	Average	0.000	0.000	0.000	0.000	0.000	0.000	0.000	0.000	0.000

Table three shows the arithmetic means of the standardized scores, for each cluster (rows) and circular economy indicator (columns). *Bold means that correlation is significant at the 0.01 or 0.05 bilateral level

In order to characterise each cluster, its behaviour was analysed with regard to (a) its own level of orientation towards CE; that is, if it was above, below, or at the average for each indicator; (b) its level of compliance with the SDGs; and (c) its level of human development measured through five indicators—Human Development Index, life expectancy at birth, expected years of schooling, mean years of schooling, and gross national income per capita.

**Fig. 1:**
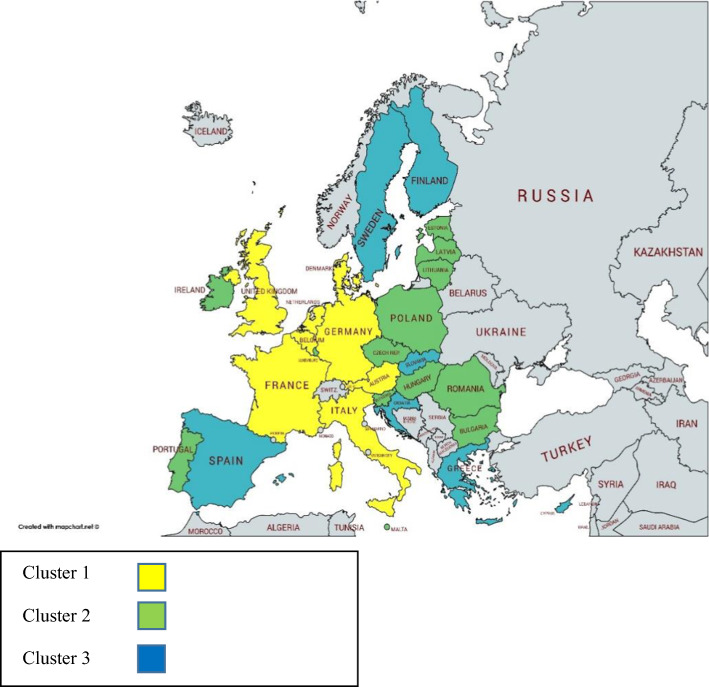
EU clusters by circular economy results

In the first group (G1) are Austria, Belgium, Denmark, France, Germany, Italy, the Netherlands, and the United Kingdom. These nations have in common that they are above average in the first five indicators of orientation towards a CE (generation of municipal waste per capita, recycling rate of municipal waste, recycling rate of overall packaging, recycling rate of e-waste, and recycling of biowaste), in 13 of the 17 SDGs (SDG 1, SDG 3, SDG 4, SDG 5, SDG 6, SDG 8, SDG 9, SDG 10, SDG 11, SDG 14, SDG 15, SDG 16, and SDG 17) and in the five human development indicators used. A second group (G2) is made up of Bulgaria, Czech Republic, Estonia, Hungary, Ireland, Latvia, Lithuania, Luxembourg, Malta, Poland, Portugal, Romania, and Slovenia, countries which are above average for six indicators of orientation towards a CE (recycling rate of municipal waste, recycling rate of e-waste, recovery rate of construction or demolition waste, circular material use rate, gross investment in tangible goods, and persons employed), for three indicators of SDG compliance (SDG 4, SDG 12, and SDG 14) and for the mean years of schooling indicator. Finally, the third group (G3) is constituted by Croatia, Cyprus, Finland, Greece, the Slovak Republic, Spain, and Sweden, which are above average in two indicators of orientation towards a CE, such as circular material use rate and persons employed, for eight indicators of compliance with the SDGs (SDG 2, SDG 3, SDG 5, SDG 7, SDG 9, SDG 10, SDG 13, and SDG 15) and for a human development level indicator such as life expectancy at birth (see Fig. 1).

Figure 1 shows the three clusters of European Union countries with differentiated results.

Regarding their orientation towards the circular economy.

Yellow colour: Cluster 1. These nations are above average in the first five indicators of orientation towards a CE.

Green colour: Cluster 2. These nations are above average for six indicators of orientation towards a CE.

Blue colour: Cluster 3. These nations are above average in two indicators of orientation towards a CE.

## Discussion

From these results, the research questions can be addressed.

As regards RQ1, which queried whether the objectives defined in the 2030 Agenda be considered homogeneous, and if they could adequately measure the concept of sustainability, it has been found that, although in some cases there are significant correlations among them (e.g. SDG 1 with SDGs, 3, 8, 13, and 16; SDG3 with SDGs 5, 6, 8, 9, 11, 12, 13, 14, 15, 16, and 17), a large part of the SDGs is not correlated with others (e.g. SDG2 with SDGs 6). As a whole, the 17 SDGs do not configure a single factor but five (SGI1, SDGI2, SDGI3, SDGI4, and SDGI5). So, it can be said that they are not homogeneous and measure at least five concepts related to sustainability. These results support the inconsistency highlighted by Spasier et al. (2017) and the conflicts among several SDGs provided by Pradhan et al. (2017)—specifically those between the economic and social goals and the environmental ones —, and the significant interactions among SDGs 1, 3, 4, 10, 12, and 13 given by Pradhan et al. (2017). Thus, there is an interconnection between the quality of life and human health goals and the environmental sustainability ones (Huttmanová et al., 2019b).

All these findings, both strong and weak correlations, seem logical, whereas the 2030 Agenda is aimed at achieving the sustainability of the planet in its three aspects of sustainability—environmental, social, and economic. Nevertheless, SDGs and sustainable development are long-term plans that can be altered by unpredictable changes, such as social advances, technological innovations, etc., or even by modifications in the dimensions of sustainability (Huttmanová et al., 2019a; Prieto-Sandoval et al., 2017), changing the current trends and transforming the future outcomes (Spasier et al., 2017).

Overall, the global initiative recognises that these Agenda 2030 goals have an interrelated nature of issues such as poverty, inequality, gender, equality, and conservation (Le Blanc, 2015). Dantas et al. (2021) identify the 17 Sustainable Development Goals as an interconnected strategy formed by goals, targets, and indicators for the promotion of sustainability practices. Furthermore, according to the UN-ESC (2017), SDGs are integrated and indivisible in the three dimensions of sustainability: economic, social, and environmental (UN-ESC, 2017).

As for RQ2, a question that seeks significant correlations between the indicators of a CE implementation in the EU and the 17 SDGs, we can state that there are significant relationships among the indicators of CE implementation in the EU and the degree of compliance with the SDGs in the European space. As has been shown, there are statistically significant relationships between SDGs 3, 6, 7, 8, 9, 10, 11, 12, 13, 14, 15, 16, and 17 with all three factors that indicate compliance with a CE in the EU. Most of these correlations are positive (SDGs 6, 8, 9, 10, 11, 12, 14, 16, and 17), but they are negative with SDGs 3 (good health and well-being), 7 (affordable and clean energy), 13 (climate action), and 15 (life on land).

Once the existence of these correlations has been evidenced, we can test the hypotheses previously formulated (see Table 5).

**Table 5:** Hypothesis testing

Hypothesis	Accepted	Rejected
H1. The CE and SDG 2 are related		**X**
H2. The CE and SDG 6 are related	**X**	
H3.The CE and SDG 8 are related	**X**	
H4. The CE and SDG 9 are related	**X**	
H5. The CE and SDG 11 are related	**X**	
H6. The CE and SDG 12 are related	**X**	
H7. The CE and SDG 13 are related	**X**	
H8. The CE and SDG 14 are related	**X**	
H9. The CE and SDG 15 are related	**X**	

Table four shows the results of the hypothesis testing

Therefore, of the nine hypotheses formulated, only one, H1, has not been positively verified; that is, we cannot affirm that there is a statistically significant relationship between the implementation of a CE and the fulfilment of SDG 2 aimed at achieving zero hunger in the EU—which in a way, seems logical because the CE does not directly aim to eradicate hunger in the European space.

Finally, we face the evaluation of RQ3, which asked if the behaviour of the 28 EU nations is homogeneous in terms of the results of the initiatives guiding the implementation of a CE in this region. The study carried out has shown that the behaviour of these European countries is not homogeneous, but it has been possible to identify three groups/clusters of countries with homogeneous results among themselves and heterogeneous results with respect to others.

The first group consists of the four most important countries in the EU according to their GDP—Germany, the United Kingdom, France, and Italy—accompanied by four others from Western Europe—Austria, Belgium, Denmark, and the Netherlands. These countries stand out with respect to many indicators both for their orientation towards a CE as well as for compliance with the SDGs and human development. Regarding their orientation towards the CE, these countries achieve the best results of the members of the EU in some EC indicators such as the generation of municipal waste per capita, the recycling rate of overall packaging, and the recycling of biowaste, and they are above average in the recycling rate of municipal waste and the recycling rate of e-waste.

The second group consists mostly of countries in Eastern Europe—Bulgaria, Czech Republic, Estonia, Hungary, Latvia, Lithuania, Poland, Romania, and Slovenia—together with some small-sized ones such as Ireland, Luxembourg, Malta, and Portugal. This group is above the European average in many indicators of orientation towards a CE: These countries achieve the best results in the following EC indicators such as the recycling rate of municipal waste, the recycling rate of e-waste, the recovery rate of construction or demolition waste, the circular material use rate, gross investment in tangible goods, and persons employed. These fall to a few indicators of SDG compliance and a single indicator of human development.

Finally, the third group is quite heterogeneous in terms of its composition because it brings together Mediterranean countries such as Spain, Greece, Croatia, and Cyprus with Nordic countries like Finland and Sweden, and one from the east, the Slovak Republic. Collectively, these countries are just above average in only two indicators of orientation towards a CE—the circular material use rate and persons employed, as well as in some indicators of compliance with the SDGs, and in one indicator of human development level.

These results indicate that, although the communications of the European Commission are addressed to all the countries that make up the EU, their own structural, social, economic, and cultural characteristics cause quite divergent results in indicators such as the generation of municipal waste per capita, the recycling rates, and the circular material use rate.

The first group is characterised by being made up of the richest countries in the EU, constituting the hardcore of the EU, with a high level of economic and social development oriented towards industry and technologies. The second block is made up of many Eastern European countries with a very strong relationship with the Soviet Union after the Second World War, all of which have had to make a great effort to modernise their economies after the disappearance of the USSR. The third block is composed by four Mediterranean countries with a clear orientation towards the economy of services and, more specifically, towards tourism—which can be an important differential element with respect to other European countries that also have an important tourism sector, although not the being most relevant for their economies—as well as two Nordic countries and one Central European.

Given the disparity of results achieved by the three groups of countries, the European Community should reflect on whether the mere publication of communications and reflections is enough for all its countries to be properly oriented towards a CE. Specifically, the European Parliament should become even more involved in the approval of mandatory standards in the field of the CE that go further and are more normative than the recommendations issued by the European Commission. These regulations may provide differential support measures so that those countries that are in more delayed phases in their policies to implement a circular model in their economy can progress and achieve their objectives in this area without falling behind from the leading countries development.

In any case, as Farmer (2020) indicates, directives must be transposed into the national law of each member state in order to be implemented. Furthermore, regulations must be directly applicable without the need for transposition into member state law, and communications have no direct legal effect, rather they establish policy directions and strategies for a topical issue, which may lead to future EU regulations or directives.

In this regard, in a recent paper by Calisto Friant et al., (2021: 337) the dichotomy between words and actions in the field of the EU’s CE Action Plan is shown. The EU has approached circularity as a means to ‘increase competitiveness, promote economic growth and create jobs while reducing environmental impacts and resource dependency’; yet, according to these authors, a ‘more holistic long-term thinking will be needed, to ensure that EU policies don't remain stuck in end-of-pipe solutions and actually bring about tangible socio-ecological change.’

Despite the efforts made by the EU Commission, we were already warned by Sachs et al. (2018) and the European Commission (2018) in 2018 of the risk that some states would not meet the targets set on reuse/recycling on municipal waste. In fact, in 2019, 14 countries were at risk of missing the 2020 municipal waste recycling target. The main reason for not complying was the lack of commitment of local authorities that did not translate the national objectives into local autonomy (Ghenea, 2020). This indicates that it is not enough to acquire commitments at the national or supranational level to advance in the transition towards a CE. Without the determined role of the agents at the local level, the desired objectives will not be achieved.

## Conclusion

The sustainability of the society in its three basic aspects—environmental, social, and economic—is one of the great challenges that humanity must face in the coming years if we want to continue maintaining the basic standards of quality of life that ensure the future of the society and its inhabitants. Faced with this challenge, the 2030 Agenda is configured as a general framework for the major sustainability objectives of the world, whose compliance will be supported by CE thanks to its economic and environmental orientation. Although the 2030 Agenda was approved for application to all countries, the ‘old’ continent, given its relative scarcity of natural resources, must lead this transformation process worldwide in order to allow its transit from a linear economy model to a new circular economy model.

The problem of defining no less than 17 SDGs is not that all of them align in a single objective. Instead, when trying to achieve results in the economic, social, and environmental aspects of sustainability, which causes them to have different orientations, all of them are valid but not always related. When the millennium goals were approved, only eight objectives had a clear orientation towards improving the living conditions of human beings and facilitated the approach to the measures to be taken. The 2030 Agenda, however, is much more ambitious and considers not only the human and social aspects but also the economic and environmental aspects. This makes the fulfilment of the actions to be undertaken much more varied and not having a common focus. So, in response to the first research question, which asked whether the objectives defined in the 2030 Agenda could be considered homogeneous, and if they could uniquely measure the concept of sustainability, the answer is that the objectives defined in the 2030 Agenda cannot be considered homogeneous, and they cannot uniquely measure the concept of sustainability.

According to the second research question, we investigated whether there are significant correlations between a CE implementation in the EU and SDGs. The relationship between the CE model and the fulfilment of the SDGs in the EU28 has been shown in this paper. At the European level, most SDGs are related to the indicators of orientation towards a CE, so it seems valid to admit that concrete actions aimed at transforming the linear economy into a CE will make it possible to comply with the 2030 Agenda. In addition, the relationships among most of the 17 SDGs lead to affirm that, either directly or indirectly, a CE will allow these objectives to be achieved in the European space by 2030. As a result, in response to the second research question, there are significant correlations between a CE implementation in the EU and SDGs.

However, despite the strong commitment that the EU is making through the different communications and recommendations issued since 2011 to promote the decisive implementation of a CE model in the common European space, it does not ensure homogeneous results for the 28 countries that have integrated a CE, and there are strong divergences among the results obtained by countries. The ‘central’ countries of the EU, the countries of the East, and finally, some Mediterranean countries, have different behaviours in terms of their transition to a CE, which can generate different positions in the challenge for the EU to adopt, in a generalised way, a CE model. All this leads us to state, as an answer to the third research question—which explored whether the behaviour of the 28 EU countries is homogeneous in terms of the results of the initiatives aimed at the implementation of a CE—that the behaviour is not homogeneous. As already stated, this difference in their performance could be partially explained by the fact that each European country has different characteristics.

This paper aims at contributing to the literature with an empirical study that relates the CE and SDGs set by the 2030 Agenda and the initiatives adopted by the European Commission since 2011. Moreover, there is a scholarly debate shedding light on three issues that, although having received certain attention in the literature lately, in our opinion need deeper study. Through our three research questions, it can be stated that the role of states or government policies is key to improving the conditions of environmental sustainability. Additionally, it is important to underline the importance of the international agreements to encourage initiatives that will exert positive impacts between the signers. If it were not for this type of agreement related to sustainability and circularity, many of the advances made at the international and national levels would not have taken place.

Therefore, a future line of research will analyse how the pandemic caused by COVID-19 will influence the sustainability in the planet. Eventually, this pandemic could be a perfect opportunity to revise the international agreements contained in the Agenda 2030 of the United Nations and the regulations developed in the EU since 2011 on the Circular Economy, to consider the effects of the former and a greater alignment among the Sustainable Development Objectives and the various indicators of the Circular Economy. Another future line of research will be oriented towards solving the apparent contradictions among the 17 SDGs. A third line will focus on establishing policies that promote the CE in those EU countries that are lagging in their achievement.

The main limitations of the present paper are: (a) both SDGs and the CE are not homogeneous constructs as they are made up of several components or factors, making them difficult to be accurately measured; (b) the delay in the publication of official data from the countries considered in this study, which constitute the basis of the empirical study, may make the results obsolete; and (c) the health pandemic caused by COVID-19 may alter government policies focused on both compliance with the 2030 Agenda and the transition to a new CE model, which stresses the importance of undertaking the first new line of research proposed in the previous paragraph.

## Appendix 1

SDG and CE variables used in the paper

**Table:**

Acronym	Variable	Type Of Relationship
SDG1V1	Poverty headcount ratio at $1.90/day (% population)	Inverse
SDG1V2	Projected poverty headcount ratio at $1.90/day in 2030 (% population)	Inverse
SDG1V3	Poverty rate after taxes and transfers, poverty line 50% (% population)	Inverse
SDG2V2	Prevalence of stunting (low height-for-age) in children under 5 years of age (%)	Inverse
SDG2V3	Prevalence of wasting in children under 5 years of age (%)	Inverse
SDG2V4	Prevalence of obesity, BMI ≥ 30 (% adult population)	Inverse
SDG2V5	Cereal yield (t/ha)	Direct
SDG2V6	Sustainable Nitrogen Management Index	Direct
SDG3V1	*Maternal mortality rate (per 100,000 live births)*	Inverse
SDG3V2	*Neonatal mortality rate (per 1,000 live births)*	Inverse
SDG3V3	Mortality rate, under-5 (per 1,000 live births)	Inverse
SDG3V4	*Incidence of tuberculosis (per 100,000 population)*	Inverse
SDG3V5	HIV prevalence (per 1,000)	Inverse
SDG3V6	*Age-standardised death rate due to cardiovascular disease, cancer, diabetes, and chronic respiratory disease in populations age 30–70 years (per 100,000 population)*	Inverse
SDG3V7	Age-standardised death rate attributable to household air pollution and ambient air pollution (per 100,000 population)	Inverse
SDG3V8	*Traffic deaths rate (per 100,000 population)*	Inverse
SDG3V9	Healthy Life Expectancy at birth (years)	Direct
SDG3V10	Adolescent fertility rate (births per 1,000 women ages 15–19)	Inverse
SDG3V11	Births attended by skilled health personnel (%	Direct
SDG3V12	Surviving infants who received 2 WHO-recommended vaccines (%)	Direct
SDG3V13	Universal Health Coverage Tracer Index (0–100)	Direct
SDG3V14	Subjective Wellbeing (average ladder score, 0–10)	Direct
SDG3V15	Gap in life expectancy at birth among regions (years)	Inverse
SDG3V16	Gap in self-reported health by income (0–100)	Inverse
SDG3V17	Daily smokers (% population age 15 +)	Inverse
SDG4V1	Net primary enrolment rate (%)	Direct
SDG4V2	Mean years of schooling	Direct
SDG4V4	Population age 25–64 with tertiary education (%)	Direct
SDG4V5	PISA score (0–600)	Direct
SDG4V6	Status (%)Variation in science performance explained by students’ socio-economic	Inverse
SDG4V7	Students performing below level 2 in science (%)	Inverse
SDG4V8	Resilient students (%)	Inverse
SDG5V1	Unmet demand for contraception, estimated (% women married or in union, ages 15–49)	Inverse
SDG5V2	Female to male mean years of schooling, population age 25 + (%)	Direct
SDG5V3	Female to male labour force participation rate (%)	Direct
SDG5V4	Seats held by women in national parliaments (%)	Direct
SDG5V5	Gender wage gap (total, % male median wage)	Inverse
SDG6V1	High-income countries: population using safely managed water services (%)	Direct
SDG6V3	High-income countries: population using safely managed sanitation services (%)	Direct
SDG6V5	Freshwater withdrawal as % total renewable water resources	Inverse
SDG6V6	Imported groundwater depletion (m3/year/capita)	Inverse
SDG7V2	Access to clean fuels & technology for cooking (% population)	Direct
SDG7V3	CO2 emissions from fuel combustion / electricity output (MtCO2/TWh)	Inverse
SDG7V4	Share of renewable energy in total final energy consumption (%)	Direct
SDG8V2	Slavery score (0–100)	Direct
SDG8V3	Adults (15 years +) with an account at a bank or other financial institution or with a mobile-money-service provider (%)	Direct
SDG8V4	Employment-to-Population ratio (%)	Direct
SDG8V5	Youth not in employment, education or training (NEET) (%)	Inverse
SDG9V1	Proportion of the population using the internet (%)	Direct
SDG9V2	Mobile broadband subscriptions (per 100 inhabitants)	Direct
SDG9V3	Quality of overall infrastructure (1 = extremely underdeveloped; 7 = extensive and efficient by international standards)	Direct
SDG9V4	Logistics performance index: Quality of trade and transport-related infrastructure (1 = low to 5 = high)	Direct
SDG9V5	The Times Higher Education Universities Ranking, Average score of top 3 universities (0–100)	Direct
SDG9V6	Number of scientific and technical journal articles (per 1,000 population)	Direct
SDG9V7	Research and development expenditure (% GDP)	Direct
SDG9V8	Research and development researchers (per 1,000 employed)	Direct
SDG9V9	Triadic patent families filed (per million population)	Direct
SDG10V1	Gini Coefficient adjusted for top income (1–100)	Inverse
SDG10V2	Palma ratio	Inverse
SDG10V3	Elderly Poverty Rate (%)	Inverse
SDG11V1	Annual mean concentration of particulate matter of less than 2.5 microns of diameter (PM2.5) in urban areas (μg/m3)	Inverse
SDG11V2	Improved water source, piped (% urban population with access)	Direct
SDG11V3	Satisfaction with public transport (%)	Direct
SDG11V4	Rent overburden rate (%)	Inverse
SDG12V1	E-waste generated (kg/capita)	Inverse
SDG12V2	Anthropogenic wastewater that receives treatment (%)	Direct
SDG12V3	Production-based SO2 emissions (kg/capita)	Inverse
SDG12V4	Net imported SO2 emissions (kg/capita)	Inverse
SDG12V5	Reactive nitrogen production footprint (kg/capita)	Inverse
SDG12V6	Net imported emissions of reactive nitrogen (kg/capita)	Inverse
SDG12V7	Non-Recycled Municipal Solid Waste (MSW in kg/person/day)	Inverse
SDG13V1	Energy-related CO2 emissions per capita (tCO2/capita)	Inverse
SDG13V2	Imported CO2 emissions, technology-adjusted (tCO2/capita)	Inverse
SDG13V4	CO2 emissions embodied in fossil fuel exports (kg/capita)	Inverse
SDG13V5	Effective Carbon Rate from all non-road energy, excluding emissions from biomass (€/tCO2)	Inverse
SDG14V1	Mean area that is protected in marine sites important to biodiversity (%)	Direct
SDG14V2	Ocean Health Index Goal-Biodiversity (0–100)	Direct
SDG14V3	Ocean Health Index Goal-Clean Waters (0–100)	Direct
SDG14V4	Ocean Health Index Goal-Fisheries (0–100)	Direct
SDG14V6	Fish caught by trawling (%)	Inverse
SDG15V1	Mean area that is protected in terrestrial sites important to biodiversity (%)	Direct
SDG15V2	Mean area that is protected in freshwater sites important to biodiversity (%)	Direct
SDG15V3	Red List Index of species survival (0–1)	Inverse
SDG15V4	Annual change in forest area (%)	Inverse
SDG15V5	Imported biodiversity threats (threats per million population)	Inverse
SDG16V1	Homicides (per 100,000 population)	Inverse
SDG16V2	Prison population (per 100,000 population)	Inverse
SDG16V3	Population who feel safe walking alone at night in city or area where they live (%)	Direct
SDG16V4	Government Efficiency (1–7)	Direct
SDG16V5	Property Rights (1–7)	Direct
SDG16V7	Corruption Perception Index (0–100)	Direct
SDG17V1	Government Health and Education spending (% GDP)	Direct
SDG17V2	High-income and all OECD DAC countries: International concessional public finance, including official development assistance (% GNI)	Direct
SDG17V5	Financial Secrecy Score (best 0–100 worst)	Inverse
CEV1	Generation of municipal waste per capita	Inverse
CEV2	Recycling rate of municipal waste	Direct
CEV3	Recycling rate of overall packaging	Direct
CEV4	Recycling rate of e-waste	Direct
CEV5	Recycling of biowaste	Direct
CEV6	Recovery rate of construction of demolition waste	Direct
CEV7	Circular material use rate	Direct
CEV8	Gross investment in tangible goods	Direct
CEV9	Persons employed	Direct

## Appendix 2

Correlation matrix between pairs of SDGs

**Table:**

	ISDG 1	ISDG 2	ISDG 3	ISDG 4	ISDG 5	ISDG 6	ISDG 7	ISDG 8	ISDG 9	ISDG 10	ISDG 11	ISDG 12	ISDG 13	ISDG 14	ISDG 15	ISDG 16	ISDG 17
ISDG1	1.0	− 0.1	**0.4(*)**	0.3	0.1	0.3	− 0.2	**0.39(*)**	0.3	0.2	0.3	− 0.1	**0.38(*)**	− 0.1	0.3	**0.54(*)**	0.0
ISDG2		1.0	0.0	0.0	0.0	** − 0.42(*)**	0.0	− 0.1	− 0.0	− 0.1	0.2	0.1	0.2	− 0.2	0.2	− 0.2	− 0.2
ISDG3			1.0	0.2	**0.39(*)**	**0.63(**)**	0.3	**0.62(**)**	**0.74(**)**	0.4	**0.70(**)**	** − 0.48(*)**	**0.40(*)**	-	**0.62(*)**	**0.77(*)**	**0.45(*)**
ISDG4				1.0	**0.63(**)**	0.1	0.2	**0.45(*)**	**0.52(**)**	− 0.1	0.2	0.1	− 0.1	**0.49(*)**	0.0	**0.52(*)**	0.1
ISDG5					1.0	0.0	**0.62(**)**	**0.53(**)**	**0.68(*)**	− 0.1	0.3	− 0.1	0.0	0.2	0.0	**0.53(*)**	**0.48(*)**
ISDG6						1.0	0.0	**0.44(**)**	**0.46(*)**	**0.53(**)**	**0.65(**)**	** − 0.38(*)**	0.2	− 0.1	**0.50(*)**	**0.61(*)**	
ISDG7							1.0	0.3	**0.42(*)**	− 0.1	0.1	0.0	0.2	− 0.3	− 0.1	0.3	0.0
ISDG8								1.0	**0.85(**)**	0.2	**0.75(**)**	** − 0.43(*)**	0.1	− 0.3	0.4	**0.90(*)**	**0.47(*)**
ISDG9									1.0	0.3	**0.79(**)**	** − 0.47(*)**	0.2	− 0.2	**0.38(*)**	**0.90(*)**	**0.51(*)**
ISDG10										1.0	0.4	− 0.2	0.3	− 0.3	0.3	0.3	0.2
ISDG11											1.0	** − 0.60(*)**	0.1	− 0.3	**0.38(*)**	**0.80(*)**	**0.49(*)**
ISDG12												1.0	− 0.3	− 0.3	**−0.47(*)**	**0.43(*)**	− 0.3
ISDG13													1.0	** 0.55(*)**	0.2	0.2	− 0.3
ISDG14														1.0	− 0.3	− 0.3	0.1
SDG15															1.0	**0.41(*)**	0.3
SDG16																1.0	**0.46(*)**
SDG17																	1.0

*The correlation is significant at 0.05 level (bilateral).

**The correlation is significant at the 0.01 level (bilateral).

## Appendix 3

Correlation matrix between SDGs and CE indexes

**Table:**

	CEI1	CEI2	CEI3	
SDG1	Correlation	0.192	− 0.162	0.053
	Sig. (bilateral)	0.327	0.411	0.789
SDG2	Correlation	− 0.282	− 0.196	− 0.134
	Sig. (bilateral)	0.146	0.318	0.495
SDG3	Correlation	0.378(*)	− 0.551(**)	− 0.046
	Sig. (bilateral)	0.048	0.002	0.816
SDG4	Correlation	0.106	0.083	0.247
	Sig. (bilateral)	0.593	0.676	0.206
SDG5	Correlation	0.150	− 0.015	− 0.120
	Sig. (bilateral)	0.445	0.938	0.545
SDG6	Correlation	0.547(**)	− 0.513(**)	0.020
	Sig. (bilateral)	0.003	0.005	0.921
SDG7	Correlation	− 0.064	0.006	− 0.387(*)
	Sig. (bilateral)	0.746	0.974	0.042
SDG8	Correlation	0.499(**)	− 0.216	0.115
	Sig. (bilateral)	0.007	0.270	0.561
SDG9	Correlation	0.512(**)	− 0.379(*)	0.016
	Sig. (bilateral)	0.005	0.047	0.935
SDG10	Correlation	0.389(*)	− 0.566(**)	− 0.121
	Sig. (bilateral)	0.041	0.002	0.539
SDG11	Correlation	0.590(**)	− 0.370	0.265
	Sig. (bilateral)	0.001	0.053	0.172
SDG12	Correlation	− 0.205	0.435(*)	− 0.162
	Sig. (bilateral)	0.296	0.021	0.411
SDG13	Correlation	− 0.254	− 0.403(*)	− 0.250
	Sig. (bilateral)	0.192	0.033	0.199
SDG14	Correlation	− 0.010	0.433(*)	0.392(*)
	Sig. (bilateral)	0.961	0.021	0.039
SDG15	Correlation	0.306	− 0.570(**)	0.006
	Sig. (bilateral)	0.113	0.002	0.977
SDG16	Correlation	0.545(**)	− 0.356	0.064
	Sig. (bilateral)	0.003	0.063	0.746
SDG17	Correlation	0.561(**)	− 0.229	0.144
	Sig. (bilateral)	0.002	0.242	0.464

*The correlation is significant at 0.05 level (bilateral).

**The correlation is significant at the 0.01 level (bilateral).
